# Antibiotic susceptibility patterns of *Staphylococcus aureus* strains isolated at the Yaounde Central Hospital, Cameroon: a retro prospective study

**DOI:** 10.11604/pamj.2019.32.103.15743

**Published:** 2019-03-05

**Authors:** Michel Kengne, Olivier Fotsing, Thérèse Ndomgue, Julius Mbekem Nwobegahay

**Affiliations:** 1 Department of Medical Microbiology and Immunology, School of Health Sciences, Catholic University of Central Africa, Yaounde, Cameroon; 2 Technicians school, Yaoundé, Cameroon; 3 Military Health Research Center (CRESAR), Yaounde, Cameroon

**Keywords:** *Staphylococcus aureus*, antibiotics susceptibility, Methicillin-resistant *Staphylococcus aureus*, methicillin-sensitive *Staphylococcus aureus*, Yaounde

## Abstract

**Introduction:**

*Staphylococcus aureus* is an important pathogen responsible for hospital and community acquired infection(s). Emerging resistance to methicillin in this organism has left physicians with few therapeutic alternatives to treat infections caused by it. This study was aimed at determining the antibiotic susceptibility patterns of *Staphylococcus aureus* strains isolated at the Yaounde Central Hospital, Cameroon.

**Methods:**

from January 2014 to November 2016, a total of 250 non repeated strains were isolated from various clinical specimens. Isolates and antibiotic susceptibility profiles were identified through standard microbiological techniques.

**Results:**

methicillin-resistant *Staphylococcus aureus* (MRSA) and methicillin-sensitive *Staphylococcus aureus* (MSSA) accounted respectively for 80% (201/205) and 20% (49/205) of the total strains isolated. MRSA strains displayed high resistance to cefoxitin (100%), cotrimoxazole (89%), vancomycin (79.7%), lincomycin (70.3%), tobramycin (72.5%), doxycycline (68.0%), kanamycin (69.7%) and erythromycin (55.7%). In contrast, a high susceptibility was observed with rifampicin (82.6%). KTG (42.3%) and constitutive MLSB (17.4%) were the most frequent phenotypes recorded.

**Conclusion:**

our results show that the carriage of acquired MRSA infections predominates in this population. Despite the noticeable multiresistance of MRSA strains to antibiotics, rifampicin remains the drugs of choice for the therapy of acquired MRSA infections in this setting. In order to slow down antimicrobial resistance, surveillance studies for antimicrobial susceptibility remains essential to identify resistance and inform policy on resistance.

## Introduction

*Staphylococcus aureus* is one of the organisms most frequently encountered in hospital and community acquired infection, especially in elderly individuals [1]; rates of *Staphylococcus aureus* infection have increased during the past 2 decades [2]. Bacteremia due to *S. aureus* has been reported to be associated with mortality rates of 15%-60% [3]. Infections with antibiotic-resistant organisms are thought to result in higher morbidity and mortality rates than are similar infections with antibiotic-susceptible strains [4]. In the early 1940s the introduction of benzylpenicillin (penicillin G) temporarily solved the problem of staphylococcal infections, but the continued use of this agent caused the selection of resistant strains, which produced penicillinase (β-lactamase) [5]. Resistance to methicillin among *S. aureus* isolates is a growing problem and poses a significant threat for effective treatment of several difficult-to-treat infections in humans [6]. High mortality rates associated to MRSA have been reported. According to the National Strategy for Combating Antibiotic Resistant Bacteria [7], there were more than 80,000 invasive MRSA infections and 11,285 related deaths in 2011. An understanding of the magnitude of the problem requires national studies to estimate the incidence of the infection and to identify antibiotic resistance. In Cameroon, there is a paucity of data on this public health issue. The aim of this study was to determine the antibiotic susceptibility patterns of *Staphylococcus aureus's* strains isolated at the Yaounde Central Hospital, Cameroon.

## Methods

**Samples collection:** this cross-sectional retroprospective study was carried out on strains of *S. aureus* isolated from pathological products of patients between January 2014 and November 2016 at the Yaounde Central Hospital (YCH). Data dealing with the retrospective survey were obtained from the hospital's data base, over a period of 28 months (January 2014-May 2016). The prospective part of the survey was conducted during a period of 6 months (June to November 2016). Participants were those attending the hospital for a bacteriological examination. Sociodemographic data were collected using a designed questionnaire. Ethical clearance was obtained from the Institutional Ethics Committee for Human Health Research of the Catholic University of Central Africa. Authorization to collect samples was obtained from YCH. Informed consent was obtained from all the study participants. Specimens were collected from both in and out patients as described by Rémic [8]. These included blood samples, pus swabs, urine samples, ear discharge, throat swab, aspirates, urethral swabs, vaginal swabs and semen.

**Isolation and identification of *Staphylococcus aureus*:** the clinical specimens were inoculated onto plates of mannitol salt agar (MSA); they were incubated at 37°C for 24h. All colonies from primary culture were purified by subculturing onto MSA medium and incubating at 37°C for 24h to 48h [9].

**Morphological characteristics:** the smear was prepared from the isolated culture on clean grease free microscopic glass slide and stained with Gram´s method of staining. The stained smear was observed under the microscope. Smear revealed Gram positive, spherical cells arranged in irregular clusters resembling to bunch of grapes.

**Biochemical examination:** biochemical tests were performed to confirm *S. aureus* using Catalase test, Coagulase test and DNase test.

**Antibiogram pattern of *S. aureus* to some antimicrobial agents:** the susceptibility of isolates to different anti-microbial agents was done by disk diffusion method using commercial disks [10] and interpreted according to EUCAST [11]. The results were recorded as susceptible(S), susceptible dose dependent (SDD) and resistant (R). Pure colonies of *S. aureus* cultures were inoculated in peptone water and incubated at 37°C to get turbidity equal to 0.5 on the McFarland scale (108 CFU/ml). A sterile cotton swab was dipped into the inoculation, and the excess was removed by pressing the swab to the sides of the tube. The entire Mueller Hinton agar surface was swabbed. The inoculation was allowed to dry for 15 min. Antibiotic discs were applied on the medium. The plates were incubated at 37°C and examined after 18-24 h. The antimicrobial agents tested were the following: cefoxitin (30μg), gentamicin (15μg), kanamycin (30 μg), tobramycin (10 μg), erythromycin (15 μg), lincomycin (15 μg), pristinamycin (15 μg), ciprofloxacin (5μg), vancomycin (30 μg), trimethoprim-sulfamethazole (25 μg), chloramphenicol (30 μg), rifampicin (5μg) and fusidic acid (30 μg).The susceptibility to methicillin was tested using a 30 μg cefoxitin disk. An inhibition halo < 25 mm was considered methicillin resistant. Macrolide, Lincosamide, Streptogramine B (MLSB) and aminoglycosides resistance phenotypes were identified as described by Courvalin *et al* [12]. The quality control of discs used was performed using *Staphylococcus aureus* (ATCC 25923).

**Data analysis:** data were entered and analyzed using SPSS version 16.0 for windows (SPSS, Inc., Chicago, IL). Discrete variables were compared using the Chi-square test. Statistical significance difference was considered at value of p < 0.05.

## Results

In total, 250 non repeated strains of *S. aureus* were included in the study. 19.6% (49/250) strains were considered as methicillin susceptible (MSSA). MRSA accounted for 80.4% (201/250) of the total strains among which 56.2% (113/201) were isolated from male and 43.8% (88/201) from female. There was no predominance of carriage of acquired MRSA infections in male compared to female (p > 0.05) as shown in *Table 1*. The frequency of MRSA according to clinical specimen is detailed in Table 2. High percentage of isolation of MRSA were obtained from pus swabs 28, 8% (58/201), vaginal swabs 25.4% (51/201) and urethral swabs 25.4% (51/59), while ear discharge gave lower percentage 0.5% (1/201). No statistical significance was found between the frequency of MRSA isolates and the clinical specimen (p > 0.05). Distribution results of the frequency of MRSA isolates according to the hospital ward (Table 3) showed that the high percentage of isolates was from out patients 49.3% (99/201). There was no significant difference (P > 0.05) between numbers of isolation in different hospital ward. In this study, MRSA isolates were found variably resistant to the antibiotics tested. High sensitivity was observed towards rifampicin (82.59%). In contrast, a high resistance of the isolates was seen with cefoxitin (100%), cotrimoxazole (89%), vancomycin (79.7%), lincomycin (70.3%), tobramycin (72.5%), doxycycline (68.0%), kanamycin (69.7%) and erythromycin (55.7%) (Table 4). Of the 201 MRSA isolates, two main resistance phenotypes were identified among the macrolides and aminoglycosides. 17.4% (35/201) exhibited the constitutive resistant (cMLSB) phenotypes and 42.3% (85/201) the Kanamycin-Tobramycin-Gentamicin (KTG) resistant phenotypes (Table 5).

**Table 1:** distribution of *S. aureus* by gender

Gender	Number of *S. aureus* isolates	Number of MRSAisolates	Percentage (%) MRSA	Statistics
**Male**	139	113	56,2	X² = 0.54, df = 2, p = 0.7
**Female**	111	88	43,8	
**Total**	250	201	100	

**Table 2:** distribution of *S. aureus* by specimen type

Specimen	Number of *S. aureus* isolates	Number of MRSA isolates	Percentage (%) MRSA	Statistics
**Throat swabs**	5	3	1,49	X² = 10.11, df = 16, p = 0.8.
**Aspirates**	9	7	3,48	
**Ear discharge**	1	1	0,50	
**Vaginal swabs**	61	51	25,37	
**Urethal swabs**	59	51	25,37	
**Pus swabs**	74	58	28,86	
**Blood**	8	5	2,49	
**Semen**	16	12	5,97	
**Urine**	17	13	6,47	
**TOTAL**	250	201	100	

**Table 3:** distribution of *S. aureus* per hospital ward

Hospital ward	Number of *S. aureus* isolates	Number of MRSA isolates	Percentage (%) MRSA	Statistics
General Medecine	82	61	30,3	Χ² = 14.38, df = 8, p = 0.07
Gynaecology	19	15	7,5	
Out patients	119	99	49,3	
Surgery	20	19	9,5	
Yaounde Emergency Center	10	7	3,5	
Total	250	201	100	

**Table 4:** susceptibility pattern of MRSA isolates to antibiotics

		MRSA (S)		MRSA (R)		MRSA (SDD)	
Antibiotics	Number of MRSA isolates	n	%	n	%	n	%
**Rifampicin**	201	166	82,59	31	15,42	4	1,99
**Ciprofloxacin**	199	129	64,82	53	26,63	17	8,54
**Chloramphnicol**	192	108	56,25	70	36,46	14	7,29
**Gentamicin**	201	108	53,73	89	44,28	4	1,99
**Pristinamycin**	195	102	52,31	74	37,95	19	9,74
**fusidic acid**	198	94	47,47	86	43,43	18	9,09
**Erithromycin**	199	75	37,69	111	55,78	13	6,53
**Kanamycin**	195	52	26,67	136	69,74	7	3,48
**Doxycyclin**	197	49	24,87	134	68,02	14	7,11
**Tobramycin**	189	43	22,75	137	72,49	9	4,76
**Lincomycin**	185	42	22,70	130	70,27	13	7,03
**Vancomycin**	197	26	47,73	157	79,70	14	7,11
**Cotrimoxazol**	200	15	07,50	178	89.00	7	3,50
**Céfoxitin**	201	0	0	201	100	0	0

**Table 5:** MRSA isolates resistant phenotypes against and aminoglycosides

Macrolides			Phenotype/ mechanism	Number of MRSA isolates	Percentage (%)	Total
Erythromycin	Lincomycin	Pristinamycin	Phenotype/ mechanism	Number of MRSA isolates	Percentage (%)	Total
**R**	R	S	MLSB constitutive	35	17,4	201
**R**	S	S	MLSB inductible	9	4,5	201
**S**	S	S	Wild type	19	9,4	201
**Aminoglycosides**						
**Kanamycin**	**Tobramycin**	**Gentamycin**	**phenotype**	**Number of MRSA isolates**	**Percentage (%)**	**Total**
**R**	R	R	KTG	85	42,3	201
**R**	R	S	KT	30	14,9	201
**R**	S	S	K	9	4,5	201
**S**	S	S	Wild type	30	14,9	201

## Discussion

*S. aureus*, one of the most common nosocomial and community-acquired pathogens has now emerged as an ever-increasing problem due to its increasing resistance to several antibiotics. This study determined the susceptibility pattern of *S. aureus* strains isolated from different clinical specimen in a tertiary hospital to provide physicians with up to date information about the local data of antimicrobial resistance of this pathogen. Of the 250 strains of *S. aureus* studied, 19.6% (49/250) strains were methicillin susceptible and 80.4% (201/250) were methicillin resistant. This study showed a high rate of MRSA which seems to be similar to findings of 72% by Njoungang [1], 76% by Gonsu [13], 65.7% by Asiimwe [14], 30.7% by Akindolire [15] and 32.5% by Eshetie [16]. As compared to present findings, lower rate of MRSA have been reported by Eibach [17], Leibler [18] and Ravensbergen [19] who found 2%, 8.3% and 10% rate respectively. It has been noticed that the proportion of MRSA has increased worldwide since the past two decades and the rate varies markedly across different countries and among hospitals of the same country [20, 21]. Improper infection prevention practices in the hospital set up, indiscriminate use of antibiotics; intravascular catheterizations, hospitalization in intensive care unit contribute in the emergence of MRSA [22]. These factors as well as the difference in the study population may explain these variations. Treatment decisions in MRSA infections require consideration of the prevalence and resistance profile of local strains. In this study, a high level of drugs resistance was found especially with vancomycin (79.70%) which is the first-line antibiotic for MRSA bacteremia treatment. This result contrasts with those reported by Karimi *et al* [23] and Swanston [24] where MRSA isolates were found susceptible to vancomycin. The high resistance of isolates to antibiotics could be due to the wide use of these drugs for the treatment of staphylococcal infections in our set up, as wide consumption of antibiotics results to the emergence of antibiotics resistant Staphylococcus species due to selective pressure.

In this study the rate of iMLSB among MSRA was found to be 4.5%. Varying prevalence rates of iMLSB have been reported in different other studies; 2.1% from Brazil [25], 8% from Jamaica [26], 18.2% from Nepal [27], 25% from Cameroon [1], 28.6% from Iran [28], 20.7% [29] and 24.3% [30] from India. Higher iMLSB prevalence of 37.5% from India [31] and 91% from Japan [32] has also been reported. A comparatively low prevalence of inducible resistance in this study could be due to the geographical variations of circulatory clones. MRSA isolates showed high rates of resistance to aminoglycoside agents. The study of resistance phenotypes of our MRSA to these drugs showed three types. 9 strains (4.5%) had a phenotype K, due to the production of the enzyme-Aminoglycoside phosphotransferase APH (3')-III. 30(14.9%) were resistant to kanamycin and tobramycin, KT is the phenotype expressed by the production of the enzyme-Aminoglycosides nucleotidyltransferases ANT (4') (4"), while 85 strains (42.3%) expressed the KTG phenotype and were resistant to the three antibiotics (kanamycin, tobramycin, gentamicin) due to the bifunctional enzyme APH (2") - Aminoglycosides acetyltransferases AAC (6') [33]. Our results are supported by those of Ida *et al* [34] and Shaw *et al* [35] who showed that most MRSA isolates seemed likely to produce AAC(6')/APH(2"), with or without ANT(4')-I, among the five kinds of aminoglycoside-modifying enzymes. In general, a beta-lactam or vancomycin often is combined with an aminoglycoside due to their synergistic effect and increased rates of killing in serious staphylococcal infections [36]. However, considering the high rates of resistance to the aminoglycosides, the addition of an aminoglycoside for the treatment of MRSA infections may be unpredictable in our set up.

## Conclusion

The burden of methicillin resistant *Staphylococcus aureus* is considerably high in this setting. The isolated strains were found to be resistant to cefoxitin, cotrimoxazole, vancomycin, lincomycin, tobramycin, doxycyclin, kanamycin and erythromycin. Rifampicin is advised for the therapy of acquired MRSA infections in this setting. Appropriate use of antibiotics remains the key to reduce the spread of multidrug resistant strains, methicillin resistant *Staphylococcus aureus* in particular. Furthermore, an antimicrobial stewardship program will be beneficial for Cameroon to promote the appropriate use of antimicrobials.

### What is known about this topic

- Bacteremia due to methicilin resistant *S. aureus* has been reported to be associated with high mortality rates;
- Resistance to methicillin among *S. aureus* isolates is a growing problem;
- Resistance to the newer antimicrobial-agents such as vancomycin are being reported.

### What this study adds

- The burden of methicillin resistant *Staphylococcus aureus* is considerably high in this setting;
- Methicillin resistant *Staphylococcus aureus* are resistant to cefoxitin, cotrimoxazole, vancomycin, lincomycin, tobramycin, doxycyclin, kanamycin and erythromyci;
- Rifampicin is the drug of choice for the therapy of acquired MRSA infections in this setting.
